# Experimental stability analysis of different water-based nanofluids

**DOI:** 10.1186/1556-276X-6-300

**Published:** 2011-04-06

**Authors:** Laura Fedele, Laura Colla, Sergio Bobbo, Simona Barison, Filippo Agresti

**Affiliations:** 1 Consiglio Nazionale delle Ricerche, Istituto per le Tecnologie della Costruzione, Corso Stati Uniti, I-35127 Padova, Italy; 2 Consiglio Nazionale delle Ricerche, Istituto per l'Energetica e le Interfasi, Corso Stati Uniti, I-35127 Padova, Italy

## Abstract

In the recent years, great interest has been devoted to the unique properties of nanofluids. The dispersion process and the nanoparticle suspension stability have been found to be critical points in the development of these new fluids. For this reason, an experimental study on the stability of water-based dispersions containing different nanoparticles, i.e. single wall carbon nanohorns (SWCNHs), titanium dioxide (TiO_2_) and copper oxide (CuO), has been developed in this study. The aim of this study is to provide stable nanofluids for selecting suitable fluids with enhanced thermal characteristics. Different dispersion techniques were considered in this study, including sonication, ball milling and high-pressure homogenization. Both the dispersion process and the use of some dispersants were investigated as a function of the nanoparticle concentration. The high-pressure homogenization was found to be the best method, and the addition of *n*-dodecyl sulphate and polyethylene glycol as dispersants, respectively in SWCNHs-water and TiO_2_-water nanofluids, improved the nanofluid stability.

## Introduction

Nanofluids are a new family of fluids, prepared by dispersing nanoparticles, i.e. particles of nanometric dimensions, in common fluids, such as water, oils or glycols. In general, the employed particles are metals, metal oxides or carbon, in different allotropic forms.

The first nanofluids were studied by Choi and Eastman in 1995 [1], to exploit their potentialities, in particular, for heat conduction applications, but until now the studies have not delved into the behaviour of these fluids. With regard to thermal engineering applications, several articles have been published showing a considerable increase of the heat transfer coefficient relative to the base fluids, due to the high thermal conductivity of the solid nanoparticles. Enhancements of up to 60% in the thermal conductivity of water-based nanofluids as per several studies were found in the literature [2,3].

Moreover, unlike the micrometric suspensions, these fluids can potentially keep a good stability over a long time, since nanoparticle aggregation and settling can be avoided. However, in fact, these two phenomena are not easy to be controlled, and they require the study of the correct combination of different variables [4]. In particular, nanoparticles often aggregate, i.e. they mix together creating clusters, because of forces of different nature, which interact amongst particles, leading to the settling down of aggregates. These two phenomena may occur independently or can be interlinked. Anyway, they involve a reduction of stability of the nanofluids and, consequently, a poor reproducibility of fluid properties.

Different experimental studies and models have been proposed to study the stability of nanofluids (e.g. [5-7]) basing on different techniques for the analysis of the stability, such as dynamic light scattering (DLS) and spectrophotometry, and considering different variables, such as nanoparticle concentration, *Z *Potential, pH and preparation method. It is also important to realize models that are able to evaluate nanoparticle aggregation and sedimentation characteristics in nanofluids. Amongst others, the most used models for the simulation of the nanoparticle behaviour within the fluid are the diffusion limited aggregation model which can be used only to describe nanoparticle aggregation [5]; the Brownian dynamics model which can be used only to describe nanoparticle sedimentation [5]; and the fractal model [8-10].

Considering the rather high discrepancy found in the published data regarding nanofluids due to the low stability of suspensions, the aim of this study is to provide successive stable fluids investigation of successively investigated as potential thermal vectors in thermal applications. New systematic data have been established, concerning the effects of different preparation methods, nanoparticle concentrations and dispersants on the stability of water-based nanofluids, obtained by dispersing titanium dioxide (TiO_2_), single wall carbon nanohorn (SWCNH) and copper oxide (CuO) nanoparticles. Up to now, several studies have been made on these three kinds of nanofluids, but the results are often discordant. The selection of a proper preparation method is essential to prevent the aggregation and sedimentation phenomena, strongly influencing the stability of the nanofluids and their thermophysical properties. For this reason, three different preparation techniques were considered, i.e. sonication, ball milling and high-pressure homogenization. Furthermore, in order to optimize the stability of the fluids, different dispersants were tested. After careful analysis of the time of the average dimension of the suspended nanoparticles by means of a DLS apparatus, Zeta potential measurements and visual observation of the suspensions, sodium *n*-dodecyl sulphate (SDS) and polyethylene glycol (PEG) were chosen as dispersants for the nanofluids based on SWCNHs and TiO_2_, respectively.

## Experimental

### Materials

Deionized water (Millipore, Billerica MA, USA, 18.2 MΩ) was used as base fluid.

The TiO_2 _nanoparticles used for the dispersions were purchased from Degussa (TiO_2_, P25), with a spherical shape and a declared 21-nm diameter.

The SWCNHs were supplied by Carbonium Srl with an estimated equivalent diameter of 100 nm.

CuO was purchased from Alfa Aesar with the indicated mean size being 30-50 nm.

The morphological characterization of nanoparticles was performed by field emission scanning electron microscopy (FE-SEM) using a SIGMA Zeiss instrument (Carl Zeiss SMT Ltd., UK).

**Figure 1:**
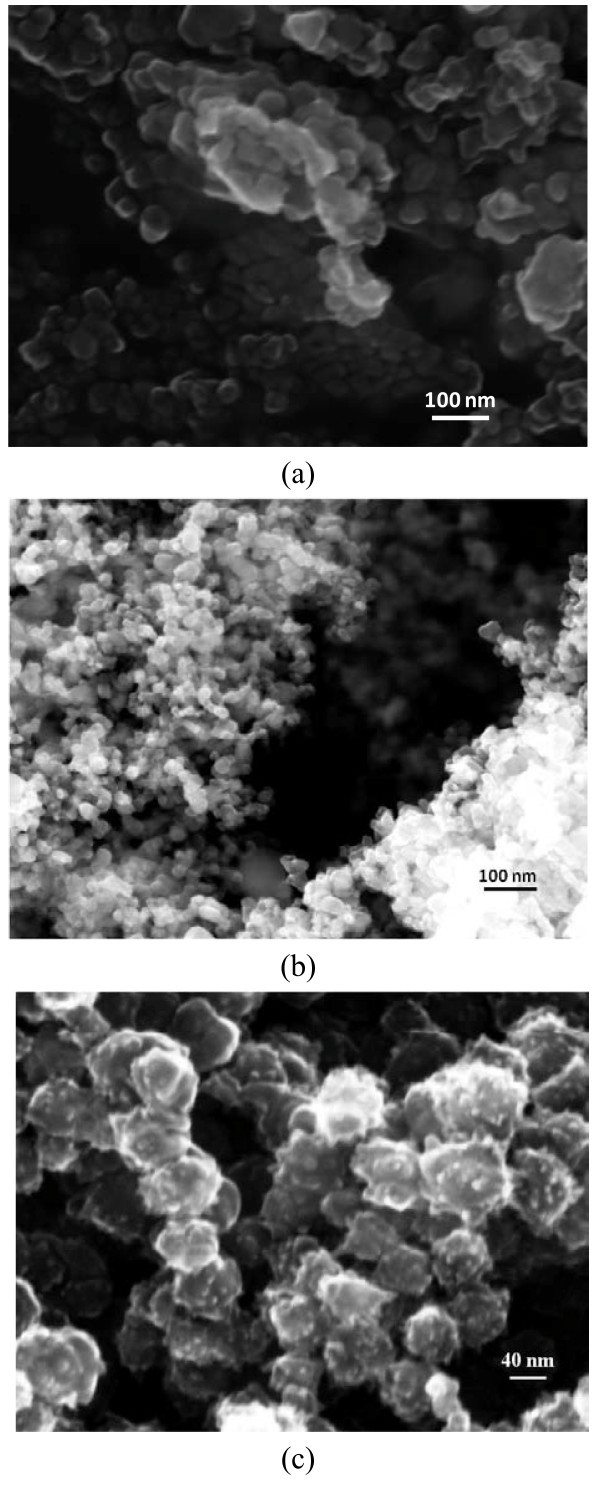
**SEM images of nanoparticles**. (a) CuO, (b) TiO_2 _and (c) SWCNH nanoparticles.

SEM images of CuO, TiO_2 _and SWCNHs are shown in Figure 1.

As dispersants, SDS (99%, Alfa Aesar), PEG 600 (Alfa Aesar), hydrochloric acid (37%, Carlo Erba) and citric acid (≥99.5%, Fluka) were tested to improve the stability of suspensions.

All the nanofluids studied in this article are summarized in Table 1, which shows the type of nanoparticle, the dispersant and the weight concentration.

**Table 1:** Water based nanofluids considered in the present work

Nanoparticles		Dispersant	
	wt.%	Compound	wt.%
CuO	0.1		
TiO_2_	0.1		
	0.01	citric acid	0.01
	0.1		
	0.1	hydrochloric acid	
	0.01	PEG	0.02
	0.1		0.2
	1		2
SWCNHs	0.1		
	0.01	SDS	0.01
	0.01		0.03
	0.1		0.1
	1		1

### Nanofluids preparation methods

The nanofluids were prepared by dispersing the nanoparticles in water by a two-step method. Three preparation techniques were compared regarding the final stability of dispersions:

• the sonication, performed at 130 W and 20 kHz for 1 h (the best solution amongst different tested sonication times) using an ultrasonic processor (VCX130, Sonics);

• the ball milling, carried out at 300 rpm for 2 h using a planetary ball mill (Pulverisette 7, Fritsch), using WC grinding bowls and 0.5-cm-diameter balls.

• the homogenization, achieved at 1000 bar using a high pressure homogenizer (GEA) with 30 passes.

### Particle size measurements

In order to evaluate the tendency of nanoparticles to aggregate and eventually sedimentate, the nanoparticle size distribution in the fluid over time was selected as control parameter. A Zetasizer Nano ZS (Malvern) was used for measuring the average dimension of the nanoparticles in solution. This instrument can detect the size from 0.6 nm to 6 μm using a DLS process. The cell is illuminated by a laser, and the particles scatter the light which is measured using a detector. The particles in a liquid move about randomly, and their speeds of movement are used for determining the size of the particle. An important feature of the Brownian motion is that small particles move quickly and large particles move slowly. There are correspondences between the size of a particle and its speed due to Brownian motion, as shown by the Stokes-Einstein equation. On the base of this physical behaviour, the Zetasizer Nano ZS measures the Brownian motion of the particles in the sample and relates this to a size based on established theories [11,12].

The particle size measured in a DLS instrument is the diameter of the ideal sphere that diffuses at the same rate of the particle being measured. All the size measurements were performed at 25°C with a scattering angle of 173°. The DLS measurements provide the size distribution using a correlation which can separate three different populations existing in the sample, showing one peak for each population. If by a measurement only one peak is found, then it means that a large majority of the particles have a diameter around the common average value.

After the nanofluids' preparation, two samples of each fluid listed in Table 1 were placed in two different measurement cuvettes. The first sample was measured almost every day for 30 days without shaking the fluid, to evaluate the size distribution changes due to natural sedimentation. The second sample was measured almost every day for 30 days after shaking the fluid, to evaluate the size distribution changes after mechanically recovering the settled particles. Each test using the Zetasizer was repeated three times, and the results shown here are the mean values of the three measurements. The measurement was always made at a constant height from the base of the cuvette. At this specific height, an average diameter was measured. For the unstable nanofluids, the diameter of the nanoparticles in the unshaken fluid decreases day after day, because of the precipitation of the bigger particles.

However, even without sedimentation, if a change in nanoparticles size occurs, indicating a nanoparticle's aggregation, then it affects the thermophysical properties of the nanofluid.

### Zeta potential measurements

Another important parameter to consider to get information on the stability of the nanofluid is the Zeta potential. In a colloidal suspension, the Zeta potential is the electric potential existing between the particle surface and the dispersing liquid at the slipping plane. The Zeta potential of nanoparticles was measured using the Zetasizer Nano (Malvern), too. This instrument uses a combination of two-measurement techniques, i.e. electrophoresis and laser Doppler velocimetry. This combination method measures the velocity of a particle in a liquid when an electrical field is applied. Then, Henry equation can be applied, knowing the viscosity and the dielectric constant of the sample. The Smoluchowski equation is used for obtaining the Zeta potential from the measured mobility for the particles in aqueous media (for high ionic strengths).

### pH measurements

Since it is known that the pH of a colloidal solution is one of the main parameters influencing the particle aggregation and the stability of the suspension, the pH of each nanofluid here considered has been measured using a pocket-sized pH meter with replaceable electrode (HANNA Instruments provided by Vetrotecnica, Padova, Italy).

## Results and discussion

In order to obtain a stable nanofluid, several water-based nanofluids were analysed and various parameters were investigated: different preparation methods, various kinds of dispersants varying both the concentration of the nanoparticles and of the dispersants. As already described, for each nanofluid, the mean size value was obtained, repeating the measurements almost every day for 30 days, both for the nanofluid stored in static mode and for the same nanofluid after mechanical shaking. Moreover, the Zeta potential measurements and the suspensions visual observation were used for analysing the nanofluid's stability.

### Comparison between different dispersion techniques

Initially, some tests on 0.1 wt.% solutions of TiO_2_, CuO and SWCNHs in water were performed, comparing the three different dispersion techniques without dispersants.

### Ball milling method

Table 2 shows nanoparticles' mean diameters at different days from their dispersions by different methods. Only 2 days are presented for the ball milling because, only after 4 days for TiO_2 _nanofluids, the nanoparticles got completely precipitated.

**Table 2:** Nanoparticles mean diameters at three different days from their preparation by three different methods, by DLS measurements on static sample

	Day from preparation	Diameter peak 1 (nm)	Diameter peak 2 (nm)	Diameter peak 3 (nm)
Ball milling method				
CuO	1	1843	5560	
	4	342		
TiO_2_	1	1281		
	4	532		
Sonication method				
CuO	1	452	4923	
	4	197		
	15	405	1407	5560
TiO_2_	1	173		
	4	154		
	15	95		
SWCNH	1	151	4830	
	4	169	4526	
	15	147	4370	
Homogenization method				
CuO	1	1248	4968	
	5	280		
TiO_2_	1	196	4936	
	5	141		
	15	117		
SWCNH	1	107		
	5	132	4714	
	15	169	4701	

All the nanofluid concentrations are 0.1 wt.%

The mean particle size obtained by ball milling was over the nanometric range (day 1). These nanofluids turned out to be unstable. In fact, from the first to the last day of measurement, the mean diameter decreased since at the constant height from the base of the cell, where the average diameter was measured, only the smaller particles remained in suspension and therefore could be detected, while the bigger ones got precipitated at the bottom of the cell. After 14 and 4 days, respectively, for CuO and TiO_2 _nanofluids, the nanoparticles, as highlighted by visual inspection, got completely precipitated and the concentration of the particles in suspension was too low to allow the measurements using the nanosizer.

Moreover, the Zeta potential was around +10 mV for CuO-water nanofluid and around 0 mV for TiO_2_-water nanofluid. These low values are typical of unstable solutions.

Considering the poor results obtained for the suspensions prepared by the ball milling process, this method was no longer tested, and other techniques were preferred.

### Sonication method

**Figure 2:**
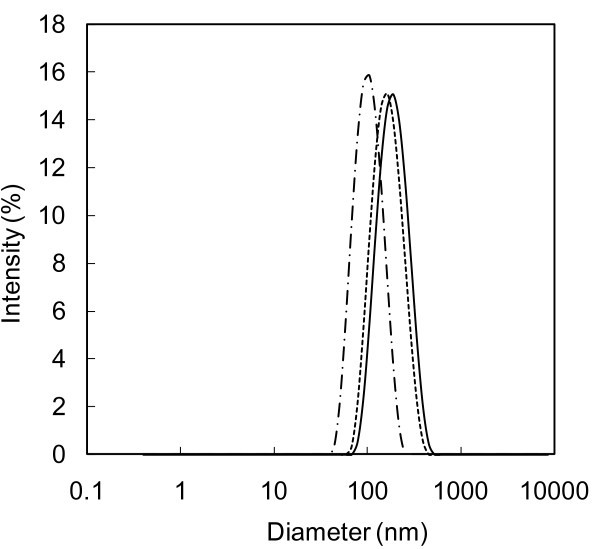
**Nanoparticles size distribution for water containing 0.1 wt.% TiO_2 _dispersed by means of the sonication method**. At (thick line) day 1, (dashed line) day 4 and (dashed-dotted line) day 15.

The mean diameter of CuO, TiO_2 _and SWCNH nanoparticles dispersed in water by sonication method are presented in Table 2, at days 1, 4 and 15. This method proved to be more effective than the ball milling method in reducing aggregates. However, in terms of stability, for CuO nanoparticles, the results are similar to those obtained by ball milling method, since they could not be measured after 15 days, because of particle precipitation, as highlighted by visual observation. Also in TiO_2_-water nanofluid, a precipitation occurred, even if being slower than with ball milling, as shown in Figure 2 which presents the nanoparticles' size distributions for water containing TiO_2 _at days 1, 4 and 15.

In SWCNHs-water nanofluid, a stable population with a 100-nm average diameter was observed, although with the presence of larger particles, with a mean diameter of approximately 4 μm, according to DLS measurements, which disappeared after 24 days, probably because of settling down.

The measured Zeta potentials were approximately +10, +50 and +35 mV for CuO, TiO_2 _and SWCNHs water-based nanofluids, respectively. Owing to the strong opacity of the SWCNHs nanofluid, it was necessary to dilute that suspension to perform the Zeta potential measurements. Considering the strong instability of TiO_2 _nanoparticles, the value obtained is in disagreement with the empirical limit of |30| mV, over which a nanofluid should remain stable.

### Homogenization method

**Figure 3:**
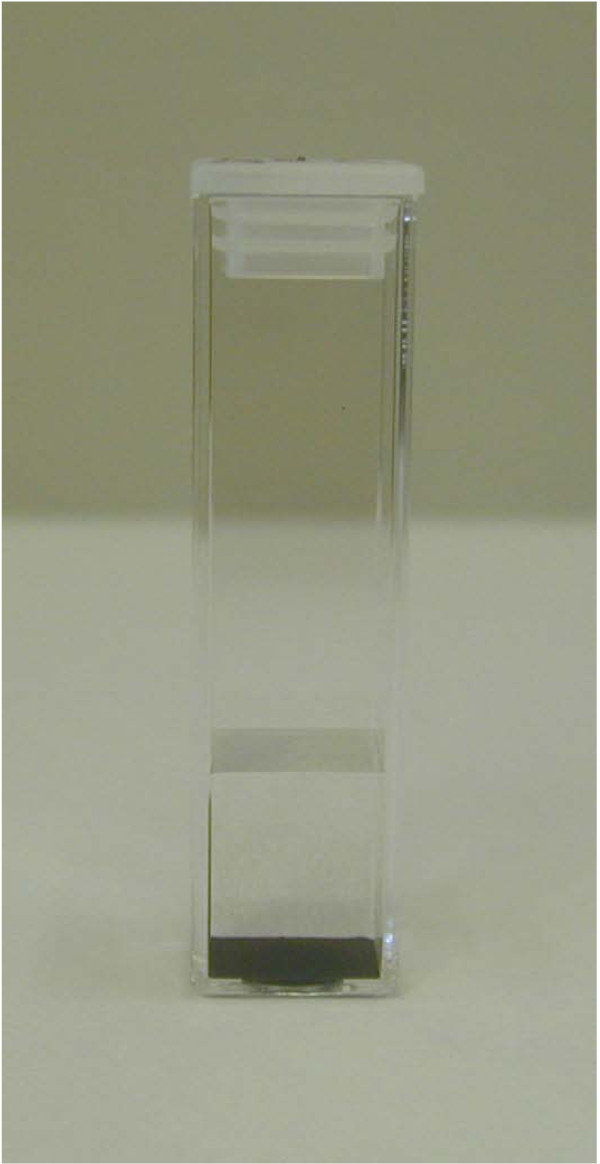
**The CuO-water nanofluid, showing precipitation just after 8 days**.

The mean diameters of CuO, TiO_2 _and SWCNH nanoparticles in water, dispersed by the homogenization method, are presented in Table 2, which shows the differences in them at days 1, 5 and 15 after preparation. The CuO-based fluid shows aggregates having mean diameters of 1 μm or more and precipitation in 8 days, as highlighted also by the visual inspection (Figure 3).

In TiO_2_-water nanofluid, all the aggregates observed on the first day precipitated after 21 days, as measured by DLS, while the other nanoparticles tended to settle down.

**Figure 4:**
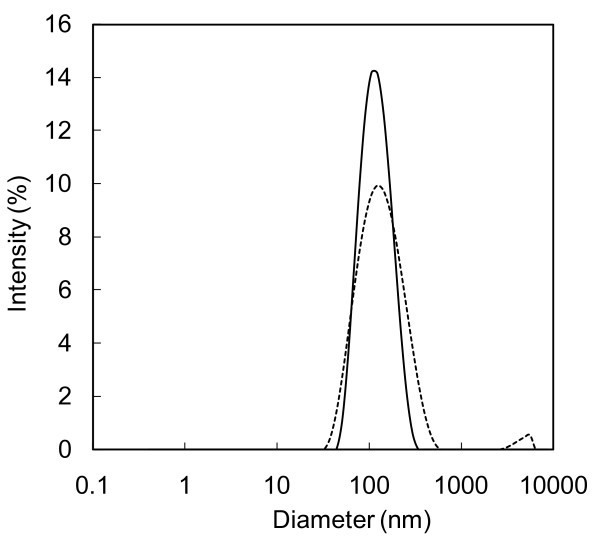
**Nanoparticles size distribution for water containing 0.1 wt% SWCNH, dispersed by means of the homogenization method.without dispersant**. At (thick line) day 1 and (dashed line) day 33.

The SWCNHs nanofluid turned out to be quite stable. In fact, the mean size measured by DLS the first day was almost constant for 33 days, as shown by Figure 4. However, from day 5, a micrometric aggregate was found, indicating a partial instability of the solution. Moreover, the mean particle size in water was slightly higher than the size measured in the powder.

The Zeta potentials for the CuO and TiO_2 _nanofluids were approximately +10 and +35 mV, respectively, while for the SWCNHs-water nanofluid, it was not possible to obtain a stable value, even after diluting the suspension.

Therefore, the homogenization process proved to be the most effective method for preparing nanofluids. However, these preliminary results pointed out that the precipitation of the CuO nanoparticles was evident even after a few days with any of the three analysed methods. For this reason, this nanofluid was no longer investigated.

At this point, in order to improve the stability of TiO_2 _and SWCNHs nanofluids, different dispersants were tested.

### Use of dispersants and acidification of the solutions

All the fluids discussed in this section were prepared with the high-pressure homogenization method, considering its superiority over the other methods. Table 3 shows nanoparticles' mean diameters and standard deviations at different days from their dispersion.

**Table 3:** Nanoparticles mean diameters and standard deviations at different days from their preparation by means of the homogenization method

	Day from preparation	Diameter peak 1 (nm)	S.D. peak 1	Diameter peak 2 (nm)	S.D. peak 2
TiO_2_/PEG (wt.%)					
0.01/0.02	1	198	0.8		
	5	166	3		
	15	268	6		
	16	159	1		
0.1/0.2	1	161	2		
	4	130	1		
	16	81	0.7		
1/2	1	132	0.7		
	5	123	1		
	15	88	0.5		
	16	86	0.4		
SWCNH/SDS (wt.%)					
0.01/0.01	1	109	0.6		
	5	131	2		
	15	106	1.4		
0.01/0.03	1	129	7		
	5	105	0.3		
	15	131	0.6	4358	427
0.1/0.1	1	101	0.4		
	5	106	0.9		
	15	115	0.6		
1/1	1	183	5		
	5	293	105	4312	158
	15	261	18	3678	1503

All the values are related to the static measurements and at most two peaks are identified

### TiO_2_-water nanofluids

Initially, two acidic solutions having pH 4-5 prepared with citric acid or hydrochloric acid were tested for the titanium dioxide-water nanofluid. In view of the potential use of these nanofluids in, e.g. hydraulic circuits, lower pH values were not considered. However, these acids were ineffective in producing stable suspensions at these pH values, since the particle precipitation was visually evident.

**Figure 5:**
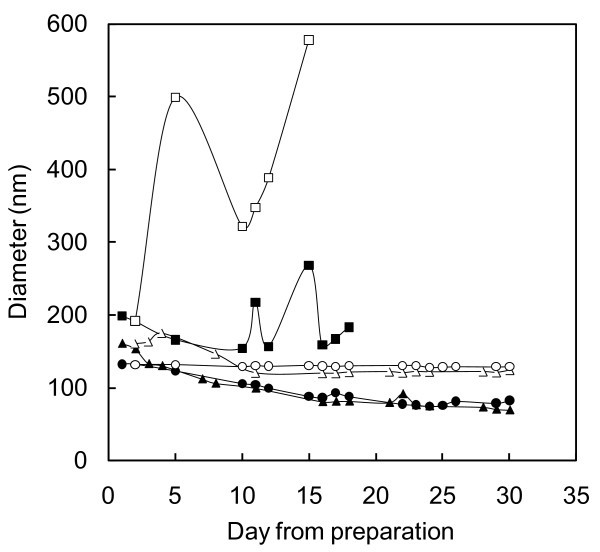
**Nanoparticles mean diameter**. Diameter in relation to the time elapsed from the day of preparation, for water containing (a) 0.01 wt.% TiO_2 _+ 0.02 wt.% PEG: (filled square) static, (open square) shaken; (b) 0.1 wt.% TiO_2 _+ 0.2 wt.% PEG: (filled triangle) static, (empty triangle) shaken; (c) 1 wt.% TiO_2 _+ 2 wt.% PEG: (filled circle) static, (open circle) shaken.

Therefore, a non-ionic dispersant, PEG 600, was investigated, based on [13]. Various concentrations of PEG and TiO_2 _were measured. The variation along time of TiO_2_-PEG nanoparticle mean diameters, with TiO_2 _at 0.01, 0.1 and 1 wt.% and PEG at 0.02, 0.2 and 2 wt.%, respectively, are shown in Figure 5.

The first nanofluid (at TiO_2 _concentration of about 0.01 wt.%) became unstable, i.e. just after 5 days, an aggregation occurred, and after 18 days, all the nanoparticles settled down (as gathered by visual observation). The irregular trend shown in the figure is probably due to the instability of the suspension.

**Figure 6:**
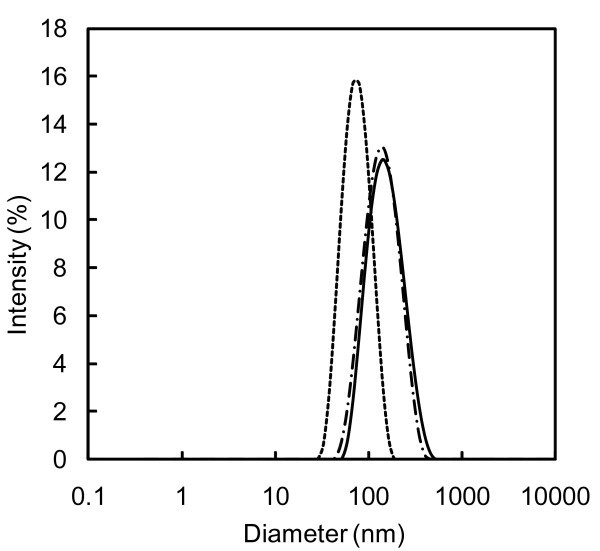
**Nanoparticles' size distribution for water containing 0.1 wt.% TiO_2 _+ 0.2 wt.% PEG**. At (thick line) day 1, (dashed line) day 30 for static and day 30 for shaken (dashed-dotted line).

On the contrary, the other samples were quite stable. In the case of static solutions, the mean size slightly decreased to around 70 nm after a few days and then it remained stable, indicating only a partial precipitation. However, after a simple mechanical shaking a mean particle size of approximately 130 nm was repeatedly recovered, suggesting the absence of further aggregation phenomena. This result is of interest because it suggests a possible application in devices where the fluids are frequently or continuously stirred, e.g. in plants with forced circulation. All the measurements provided average diameters higher than the 21 nm of the base powder, but the aggregates grew just after preparation, keeping nanometric and constant dimensions even after 30 days. In order to highlight this behaviour, Figure 6 represents the nanoparticle size distribution for water-TiO_2 _at 0.1 and 0.2 wt.% PEG. After 30 days, while the static sample shows a smaller average diameter than at the first day, the shaken nanofluid gives the same value, i.e. no further aggregation was detected.

The measured Zeta potential was +40 mV for the nanofluids containing 1 and 0.1 wt.% TiO_2_, supporting their non-aggregating tendency, while the values obtained for the 0.01 wt.% TiO_2 _fluid were not stable. The PEG:TiO_2 _= 2:1 ratio turned out to be effective, but further research is needed to optimize nanoparticle and dispersant concentration as a function of their application.

### SWCNHs-water nanofluids

SWCNHs-water nanofluids with SDS as dispersant were tested in several concentrations. An anionic dispersant was chosen based on [14]. The investigated fluids were

• water +0.01, 0.1 and 1 wt.% SDS at 0.01, 0.1 and 1 wt.% SWCNHs, respectively;

• water +0.01 wt.% SWCNHs +0.03 wt.% SDS.

**Figure 7:**
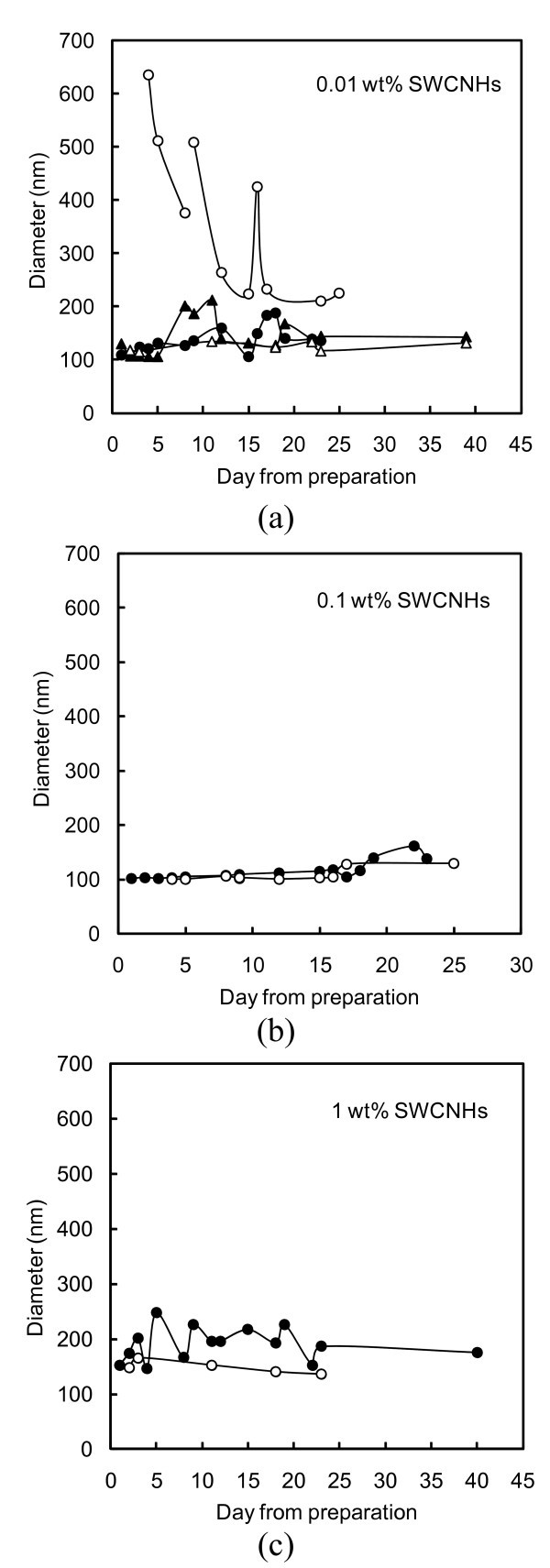
**Nanoparticles' mean diameter**. Diameter in relation to the time elapsed from the day of preparation, for water containing (a) 0.01 wt.% SWCNHs + 0.01 wt.% SDS: (filled circle) static, (open circle) shaken; 0.01 wt.% SWCNHs + 0.03 wt.% SDS: (filled triangle) static, (empty triangle) shaken; (b) 0.1 wt.% SWCNHs + 0.1 wt.% SDS: (filled circle) static, (open circle) shaken; (c) 1 wt.% SWCNHs + 1 wt.% SDS: (filled circle) static, (open circle) shaken.

Figure 7 represents the mean particle diameters as a function of time for the nanofluid in static mode and for the same nanofluid after mechanical shaking.

Water-SWCNHs containing 0.01 wt.% SDS formed aggregates, which are visible in Figure 7a in the upper curve relative to the shaken nanofluid. In order to improve the stability of this suspension, a higher SDS:SWCNHs ratio was tested. The result is shown in the same figure with triangle, where the suspension containing 0.01 wt.% of SWCNHs and 0.03 wt.% of SDS showed a very stable behaviour for 39 days, keeping a mean diameter of about 120 nm.

Water-SWCNHs containing 0.1 wt.% SDS (Figure 7b) shows a constant diameter around 100 nm, i.e. a value very similar to the one measured for the powder, for both the static and stirred sample even after 25 days, suggesting a good stability of the fluid.

Analogous behaviour was shown by water-SWCNHs containing 1 wt.% SDS (Figure 7c), though the mean diameter of nanoparticles was about 180 nm.

The measured Zeta potential was around -40 mV, negative as expected in the case of anionic dispersant [6,14], for all the studied SWCNHs-nanofluids, supporting their non-aggregating tendency. Owing to the strong opacity of the solutions at 0.1 and 1 wt.%, they were diluted to perform the Zeta potential measurements.

In conclusion, the water-based nanofluids containing SWCNHs and SDS proved to be very stable and further investigation on their properties is underway.

## Conclusion

Water-based nanofluids, obtained by dispersing titanium dioxide, SWCNH and copper oxide nanoparticles, were investigated. By using a DLS apparatus, different preparation techniques, i.e. ball milling, sonication and high pressure homogenization, were compared. In fact, size measurements can detect the mean diameter distribution variation along time and therefore the nanoparticles have the tendency to settle down. Moreover, Zeta potential measurements indicate the nanoparticles' tendency to aggregate. All these measurements, coupled with the visual observation of the suspension, permitted a stability analysis of the nanofluids.

The ball milling method turned out to be the worst one to obtain a stable nanofluid, while the homogenization method was the more effective and, therefore, it was selected to prepare the fluids in which the dispersants were added.

PEG and SDS were found to be good dispersants for the nanofluids based on TiO_2 _and SWCNHs, respectively. Water-TiO_2 _at 0.1 and 1 wt.% and with a PEG:TiO_2 _= 2:1 ratio showed a fairly good stability when the fluids are stirred, suggesting their applications in systems where they are always kept in motion. Water containing 0.01, 0.1 and 1 wt.% SWCNHs and 0.03, 0.1 and 1 wt.% SDS, respectively, proved to be very stable even in static mode for at least 25 days.

Therefore, this study demonstrated the feasibility of stable nanofluids by controlling various variables. Further development is need for the optimization of the dispersant concentration and the study of the properties of these fluids.

## Abbreviations

DLS: dynamic light scattering; FE-SEM: field emission scanning electron microscopy; PEG: polyethylene glycol; SDS: sodium *n*-dodecyl sulphate; SWCNHs: single wall carbon nanohorns.

## Competing interests

The authors declare that they have no competing interests.

## Authors' contributions

SBarison and FA carried out the nanofluid preparation step. LC performed the DLS and Z potential measurements. LF and SBobbo conceived the study and analyzed the results. All authors read and approved the final manuscript.
